# Your Brain on Art: Emergent Cortical Dynamics During Aesthetic Experiences

**DOI:** 10.3389/fnhum.2015.00626

**Published:** 2015-11-18

**Authors:** Kimberly L. Kontson, Murad Megjhani, Justin A. Brantley, Jesus G. Cruz-Garza, Sho Nakagome, Dario Robleto, Michelle White, Eugene Civillico, Jose L. Contreras-Vidal

**Affiliations:** ^1^Office of Science and Engineering Laboratories, Division of Biomedical Physics, Center for Devices and Radiological Health, U.S. Food and Drug AdministrationSilver Spring, MD, USA; ^2^Laboratory for Non-Invasive Brain Machine Interfaces, Department of Electrical and Computer Engineering, University of HoustonHouston, TX, USA; ^3^American ArtistHouston, TX, USA; ^4^The Menil CollectionHouston, TX, USA

**Keywords:** EEG, machine learning, functional connectivity (FC), aesthetics, freely moving

## Abstract

The brain response to conceptual art was studied with mobile electroencephalography (EEG) to examine the neural basis of aesthetic experiences. In contrast to most studies of perceptual phenomena, participants were moving and thinking freely as they viewed the exhibit *The Boundary of Life is Quietly Crossed* by Dario Robleto at the Menil Collection-Houston. The brain activity of over 400 subjects was recorded using dry-electrode and one reference gel-based EEG systems over a period of 3 months. Here, we report initial findings based on the reference system. EEG segments corresponding to each art piece were grouped into one of three classes (complex, moderate, and baseline) based on analysis of a digital image of each piece. Time, frequency, and wavelet features extracted from EEG were used to classify patterns associated with viewing art, and ranked based on their relevance for classification. The maximum classification accuracy was 55% (chance = 33%) with delta and gamma features the most relevant for classification. Functional analysis revealed a significant increase in connection strength in localized brain networks while subjects viewed the most aesthetically pleasing art compared to viewing a blank wall. The direction of signal flow showed early recruitment of broad posterior areas followed by focal anterior activation. Significant differences in the strength of connections were also observed across age and gender. This work provides evidence that EEG, deployed on freely behaving subjects, can detect selective signal flow in neural networks, identify significant differences between subject groups, and report with greater-than-chance accuracy the complexity of a subject's visual percept of aesthetically pleasing art. Our approach, which allows acquisition of neural activity “in action and context,” could lead to understanding of how the brain integrates sensory input and its ongoing internal state to produce the phenomenon which we term aesthetic experience.

## Introduction

Working from overlapping, but not necessarily congruent, sets of motivations, scientists, and artists alike seek an understanding of art appreciation and aesthetic judgment. From a neuroscience perspective, we might think of an aesthetic judgment or response as an integrative phenomenon consisting of processing of sensory input, internal decision-making, and internally generated emotional response. Early theories of aesthetic preference were mainly rooted in the psychological sciences (Martin, 1906; Bullough, 1957; Pratt, 1961); however, more recently, researchers have developed more physiologically-rooted models of aesthetic processing in an attempt to explain the biological underpinnings of aesthetic phenomena (Leder et al., 2004; Bullot and Reber, 2013). The first step in these models is the perceptual analysis of the object being viewed (Leder et al., 2004; Bullot and Reber, 2013). The observer is said to take in all physical features of the object (color, texture, shape, etc.) and complete initial semantic processing of the symbolic and narrative structures within the artwork (Bullot and Reber, 2013). In one model by Bullot and Reber, it is said that the elicitation of aesthetic emotion also occurs during this first step in the art appreciation process (Bullot and Reber, 2013).

Modern wearable neurotechnology brings the opportunity to advance the quantitative understanding of the aesthetic experience by studying real-time effects of the particular subset of sensory experiences that we designate as “art” on the brains of viewers experiencing it in an unconstrained, “real-world” environment. In the current study, we used non-invasive scalp electroencephalography (EEG) to measure the brain activity of museum-goers as they viewed an exhibit entitled “*The Boundary of Life is Quietly Crossed”* by conceptual artist Dario Robleto at the Menil Collection in Houston, TX (www.menil.org). In addition to brain and location data for the subjects, we surveyed them on the visual and emotional appeal of the different pieces viewed. This combination of data modalities allowed the linking of physiology to visual complexity and emotional content during the first steps of the art appreciation process.

Prior studies have deployed EEG to probe the neural basis of emotional and aesthetic stimuli and attempted to use brain activity to accurately predict the emotional state of subjects (Bradley et al., 2007; Murugappan et al., 2009; Wang et al., 2011; Lee and Hsieh, 2014). However, nearly all of these studies have been performed in a controlled experimental environment, which limits the range of tasks that participants can perform. Wired EEG systems can limit the study of dynamic brain networks in real behavioral contexts, due to movement restrictions which may be required to obtain relevant data. This is a critical flaw, as the models for aesthetic appreciation previously mentioned emphasize the effect that context can have on the experience of the observer (Leder et al., 2014). These theories state that an observer needs to have an “aesthetic attitude” in order to manifest aesthetic responses (Cupchik and László, 1992). A recent study examining how context (laboratory vs. non-laboratory environments) modulates the relation between art experience and viewing time showed that participants viewing art in a museum setting were more likely to enjoy the exhibit and view each piece longer (Brieber et al., 2014). In light of these insights, the collection of data from freely behaving subjects in the museum environment as opposed to a laboratory setting is an important feature of this study protocol.

With the real-world protocol employed here, we have addressed two major questions. First, can we resolve differences in brain activity when participants are viewing and experiencing complex pieces of art in a relatively uncontrolled setting? Studies of the brain response to images have shown response features sensitive to complexity, with one study in particular showing perceptual organization of an object reflected earlier in the late positive potential after the initial stimulus, with more positive and negative extremes occurring with a more complex stimulus or image (Bradley et al., 2007). Other studies have reported changes in oscillatory brain patterns in the 4–35 Hz range while participants viewed complex images in a memory task (Palomäki et al., 2012).

Second, can we map functional neural networks engaged during aesthetic perception and judgment? Many regions of the brain are associated with the processing of emotional and visually complex stimuli. EEG studies have reported changes in frontal and posterior brain areas (Bradley et al., 2007), while neuroimaging studies have shown that the orbitofrontal cortex (OFC) is activated during situations in which the subject is required to make appraisals of the quality of objects (e.g., artwork; Kringelbach, 2005; Wallis, 2007; Brown et al., 2011). A better understanding of the functional brain networks involved with emotional and aesthetic processing could lead to advancements in neurotechnologies intended to restore or enhance sensory processing in neurologically-impaired persons (Molina et al., 2009; Kashihara, 2014).

To address these two questions, we have collected and analyzed brain activity from a diverse group of individuals as they experienced an exhibit at the Menil Collection in Houston, Texas. Here we report initial findings from (1) quantitative differences in EEG signals across subjects of different age and gender, (2) patterns of brain activity associated with emotionally stimulating and aesthetically pleasing pieces, and (3) putative networks engaged in the perception and judgments of art stimuli.

## Materials and methods

### Subjects

Four hundred and thirty one subjects participated voluntarily in this study. Anonymous Informed Consent was approved by the University of Houston Institutional Review Board to minimize the disruption of the museum environment and protect the privacy of the participants. Potential participants were approached as they walked through the museum halls and asked if they wanted to participate in a study in collaboration with the University of Houston and the conceptual artist, Dario Robleto. Potential participants were given a brief overview of the study, how EEG works, and the expected goals and methods of data analysis. If participants agreed to participate, they were fitted with an EEG headset. Here, we report findings from 20 subjects who donned the reference gel-based EEG system. Findings from remaining subjects that wore dry-electrode EEG systems will be reported elsewhere.

### EEG headset

Twenty subjects using the 32 channel Brain Products actiCAP (BP gel) along with the Brain Amp DC amplifer were used in the analyses. BP gel is an active gel-based EEG system (actiCap system, Brain Products GmbH, Germany). The electrodes were labeled in accordance with the extended 10–20 international system. EEG data were online referenced to channel FCz on the superior region of the scalp. In addition, two channels from the posterior peripheral channels (PO7 and PO8) were used to collect electrooculography (EOG) from below and on the temple of the right eye. All data were collected wirelessly in the DC mode at a sampling rate of 1000 Hz. The BrainVision software was used for all data collection, including manual tracking annotations.

### Experimental protocol

#### Description of task

Participants in the study were fitted with the wireless EEG head-set. Once the quality of the signal was checked, the participants were asked to sit in a relaxed, comfortable position with their eyes open and facing a white wall for 1 min to acquire baseline data devoid of aesthetic content. Participants were asked to proceed to Dario Robleto's exhibit room (see Supplementary Figure 1). No restrictions on the participant's movements, behavior, duration of the visit, or thoughts were dictated. Annotations within the EEG file were manually entered as the subject arrived at a specific piece. Once the participant finished viewing the exhibit, they were asked to complete a brief questionnaire (see Supplementary Material) to ascertain the preferred pieces according to aesthetic appeal and emotional stimulation, as well as to collect basic demographic information.

#### Description of the exhibit

The entire exhibit was contained in a small room (20 × 25 ft) with various cases and windows containing all pieces throughout the room. A brief description of the intent and purpose of the exhibit “*The Boundary of Life is Quietly Crossed*” from the artist Dario Robleto, a conceptual artist based in Houston (www.dariorobleto.com), is given below:

From the first time one human placed her or his ear to another's chest, the mysteries of the movements and sounds of the heartbeat and pulse have shaped much of the human imagination around art, religion, and science. But for almost all of recorded history, this relationship to the heart as movement and sound has been an ephemeral, fleeting event. There was simply no way to objectively record the ceaseless activity of the heart. Most physicians through history have had to rely on the inherent subjectivity and fragility of human hearing and memory to diagnose and recall the heart. Without a means to permanently record the heart, it would remain mostly elusive. However, in the mid Nineteenth century, ambitious and ingenious attempts by scientists to first record and visually register these movements initiated a sequence of events that are part of a widely unknown history of the human heartbeat. Through a series of sculptures, installations, sound compositions and a book, the project entitled *The Boundary of Life is Quietly Crossed* will creatively reconstruct this forgotten history by identifying key moments in its narrative.

Because of ancient cultural understandings of the heart, any history of the heartbeat is also by necessity a history of emotions. The installation also seeks to explore how art, religion, and science have historically addressed this relationship. The project is anchored around three key narratives of the heartbeat: the first attempt to visually and audibly record the human pulse in real time, the EKG and EEG recordings of a 27-year-old woman who had just fallen in love and which were placed aboard two space probes now heading into interstellar space, and recent developments within artificial heart technology that suggest the only way to advance is by letting go of the pulsatile, or “beating,” actions of the heart. In each of these narratives there are profound implications riding on the meaning we place in the recording of the heart. They each challenge and question fundamental associations we tie to the human heart: life, death, personal identity, continuity of memory, the physical site of emotion, authenticity, creativity, and spirituality, to name a few.

The pieces in the exhibit were grouped together to form eight main pieces on which the proceeding analyses were based. Table 1 gives a brief description of each piece, as well as a photograph of the piece in the exhibit space. Permission from the artist was obtained to publish images of his work in this journal article. For those works that were not brought in by the artist, permissions to publish were obtained from the Artists Rights Society (ARS) and the Menil Collection.

**Table 1:** **Description of pieces included in the exhibit titled “*The Boundary of Life is Quietly Crossed*” by conceptual artist Dario Robleto**.

**Piece**	**Description**	**Image**
1	*Things Placed In the Sea, Become the Sea, 2013–2014*. Sea urchin shells and spines cast and coated with hand-ground and melted vinyl records salvaged from the deep sea, stretched audiotape recordings of various probe and heartbeat signals.	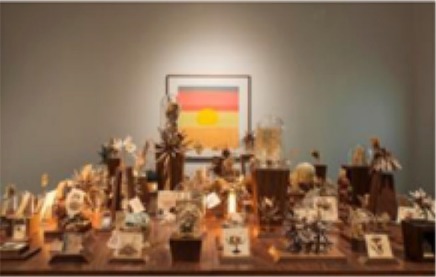
2	*Fossilhood Is Not Our Forever, 2013–2014*. Fossilized prehistoric whale ear bones salvaged from the sea (1–10 million years), stretched audiotape of three centuries of human heartbeat recordings (1865, 1977, 2014).	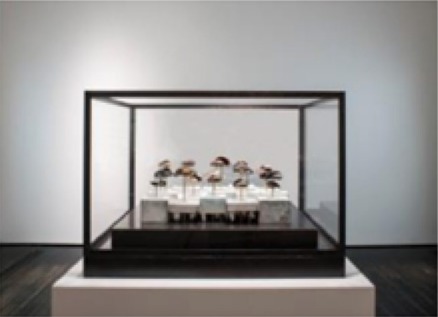
3	Several individual pieces were contained in this window. The content of this window display focused on the earliest human heart beat recordings, as well as the oldest-born heart from which a heartbeat was recorded.	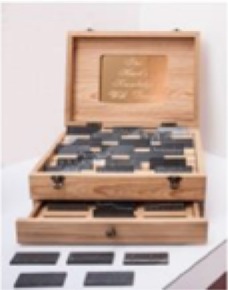
4^*^	*Andy Warhol, Sunset, 1972. Silkscreen with various colors. The Menil Collection, Houston, Gift of the artist*.	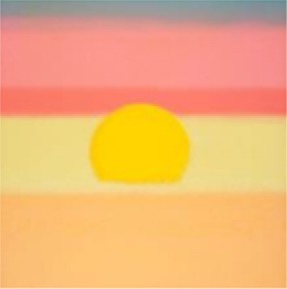
5^*^	*Several individual pieces by various artists were contained on this wall. Photographs and paintings with themes of outer space and space exploration were included*.	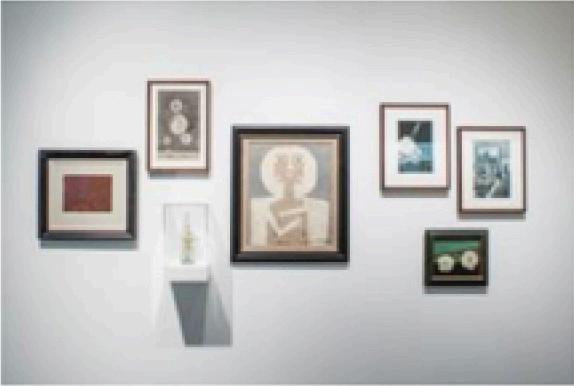
6	*Max Ernst, Undulating Earthquake (Tremblement de terre ondulatoire), 1928. Oil on canvas mounted on masonite. The Menil Collection, Houston*.	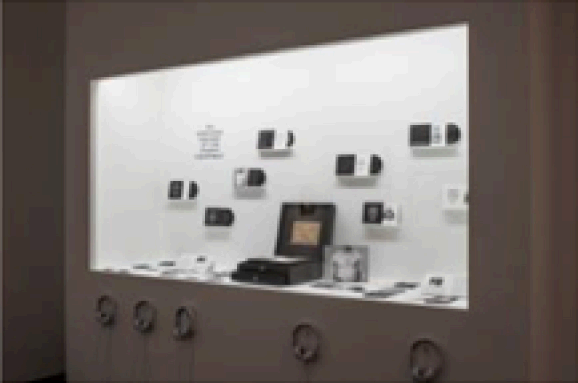
7^*^	*Max Ernst–Undulating Earthquake*	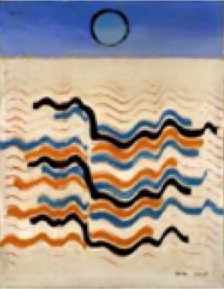
8	Several individual pieces were contained in this window. The content of this window display focused on prints of cerebral pulses, observation and depiction of emotions, and several images of devices used to measure changes of blood flow in different regions of the body.	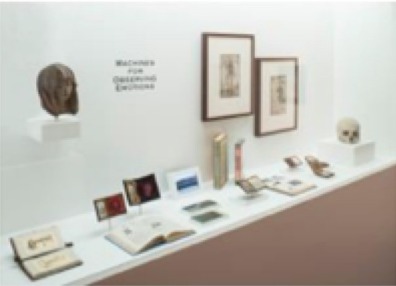

Descriptions were provided by the artist and the Menil Collection. Pieces 4, 5 and 7 (

* *) are part of the Menil's permanent collection, and were selected by Dario Robleto to be showcased in his exhibit*.

*Installation photos by Paul Hester*.

### Data pre-processing

**Figure 1:**
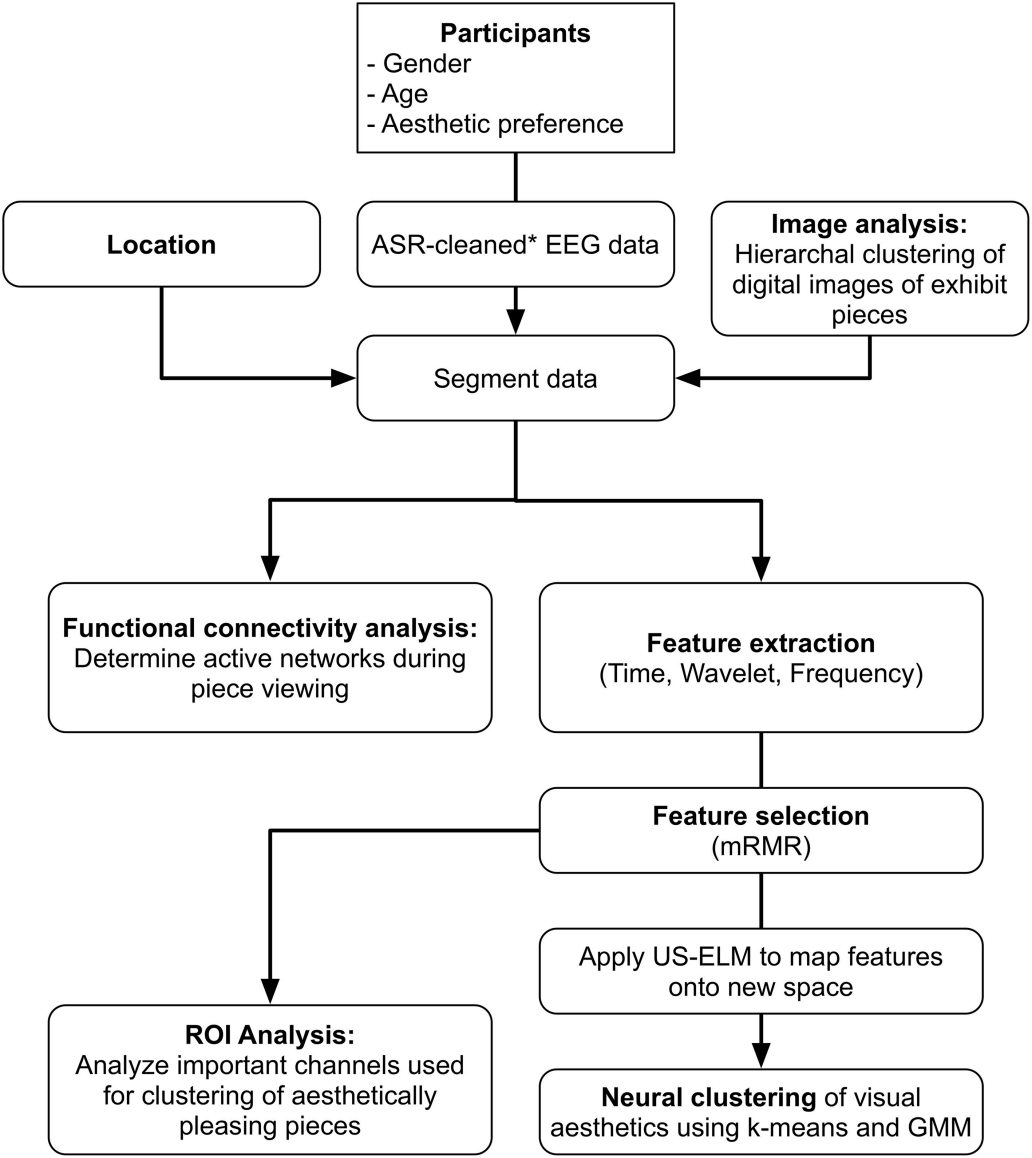
**Data processing flow chart**.

All data analysis, clustering, and functional evaluation of brain signals were performed offline using custom software developed in Matlab 2014a (Mathworks, Natick, MA). Figure 1 shows a flowchart of the data preprocessing and analysis.

The raw EEG was high-pass filtered using a zero-phase lag 8th order Butterworth filter at 0.1 Hz to remove DC shifts (Garipelli et al., 2013). Next, bad channels were identified using a correlation method (Mullen et al., 2013), as follows. Briefly, the entire time series for each subject was divided into 2-s time windows. The correlation of a given channel, X, with every other channel in that time window was calculated. If the correlation value of 80% of the channels was less than the user-defined correlation threshold, channel X would be marked “bad” for that 2 s time window. If 50% of the time windows for a given channel were marked “bad,” the channel was rejected and removed before further analysis. Given evidence that shows facial muscle activity mostly contribute EMG artifacts to peripheral sensors, the peripheral channels most susceptible to motion and myoelectric scalp artifacts were removed from the BP gel system (FP1, FP2, F7, F8, T7, T8, TP9, TP10, P7, P8, PO9, PO10; Goncharova et al., 2003).

Artifact subspace reconstruction (ASR) was used to remove artifactual components (i.e., eye movements, subject motion, cable pulling, electrode popping, etc.) from the high-pass filtered EEG data (Mullen et al., 2013). ASR is an automated artifact rejection method available as a plug-in through EEGLAB (Delorme and Makeig, 2004) that uses a sliding window technique to identify segments of EEG data corrupted with artifacts. The algorithm first identifies regions of clean EEG data and computes an un-mixing matrix from those data based on the geometric median. Within each sliding window, principal subspaces which exceed a defined threshold (in terms of standard deviations) compared to the calibration EEG data are reconstructed using the un-mixing matrix. For this study, a window length of 0.5 s and a threshold of three standard deviations were used. Given the potential for eye movement-induced increases in power of relevant EEG frequency bands, the ability of ASR to remove eye movement artifacts was compared to Independent Component Analysis (ICA). Results indicated that ASR performed similarly to ICA when identifying and correcting eye movements. The data were then re-referenced using the common average reference (CAR) (Delorme and Makeig, 2004; Garipelli et al., 2013) and segmented according to the piece the subject was viewing.

### Image analysis

Features pertaining to luminance, texture, gradient, and composite features (Gunsel et al., 2005; Lombardi, 2005) were computed and used in a hierarchal clustering algorithm to identify pieces with similar visual aspects. A brief description of each image feature is given below:

- Luminance: Luminance is roughly analogous to the “brightness” of an image. The luminance component of a color image defined in RGB space is computed by
  (1) $$\begin{array}{l} Y=0.299R+0.587G+0.114B \end{array}$$
  After transforming the image to grayscale using Equation (1), the mean, standard deviation, kurtosis, and skewness of the luminance values were computed. The percentage of dark pixels, defined as pixels with an intensity value of < 64 (Gunsel et al., 2005), was also computed by applying the following equation:
  (2) $$\begin{array}{l} \text{\% of dark pixels}=\frac{Number\;of\;dark\;pixels\;in\;the\;image}{Total\;number\;of\;pixels} \end{array}$$
- Texture: This feature has been shown to be one of the most effective types of features in classification of artistic style, as it more closely approximates brushwork than any other type of feature (Lombardi, 2005).
  A Gabor filter provides one option for describing the texture of artwork. Gabor filters transform grayscale images into coefficients which correlate with a perception of texture over multiple scales and orientations. The mean and variance of the coefficients were extracted from four scales and four orientations.
- Gradient: To extract edge information, gradient maps that measure the rate of change in intensities across an image were employed.
- Composite features: Each image was segmented into *n* identically sized blocks. After the partition of the image into identical blocks, the deviation of average gray level acquired within each block from the average gray level acquired within the entire image was computed. This feature provides invariance to changing lighting conditions and variability in the image.

### Feature extraction

Features were extracted from the EEG data using the first 5 s after the subject's annotated arrival at a given piece in the exhibit. Since the subjects were unconstrained in their thoughts and movements, it is difficult to determine the exact time at which the subject fixated on the piece. Therefore, the first 5 s of data were further divided into 1-s segments. Using each of these 1-s segments, several features were calculated in the frequency, wavelet, and time domains to characterize the properties, information content or distribution of the EEG signal as we are interested in understanding what the aesthetic experience implies in neural terms. In the context of the current work, “frequency domain” refers to the calculation of features based on spectral content of the signal extracted using the power spectral density multi-taper method, “wavelet domain” refers to the calculation of features based on spectral content of the signal extracted using the wavelet transform, and “time domain” refers to the calculation of features from the voltage amplitude data. Five frequency bands of interest were used in the feature calculation: delta (1–4 Hz), theta (4–7.5 Hz), alpha (8–12 Hz), beta (15–25 Hz), and gamma (30–50 Hz). For each channel and each frequency band, power, standard deviation, and Shannon entropy (Murugappan et al., 2009) were calculated in the frequency domain for a total of 15 features per channel (i.e., $\frac{5\;frequency\;bands}{\;channel}\times \frac{3\;features}{\;frequency\;band}=\frac{15\;features}{channel}$). A Morlet wavelet was used to calculate the same features for each channel and frequency band in the wavelet domain for a total of 15 features per channel. In the time domain, the kurtosis, standard deviation, and maximum value of the signal amplitude, as well as Shannon entropy were calculated after the signal was filtered in each of the five frequency bands stated above. These values were also calculated on the 1-s time series without any additional filtering for a total of 24 features per channel in the time domain.

### Feature selection and unsupervised clustering

**Figure 2:**
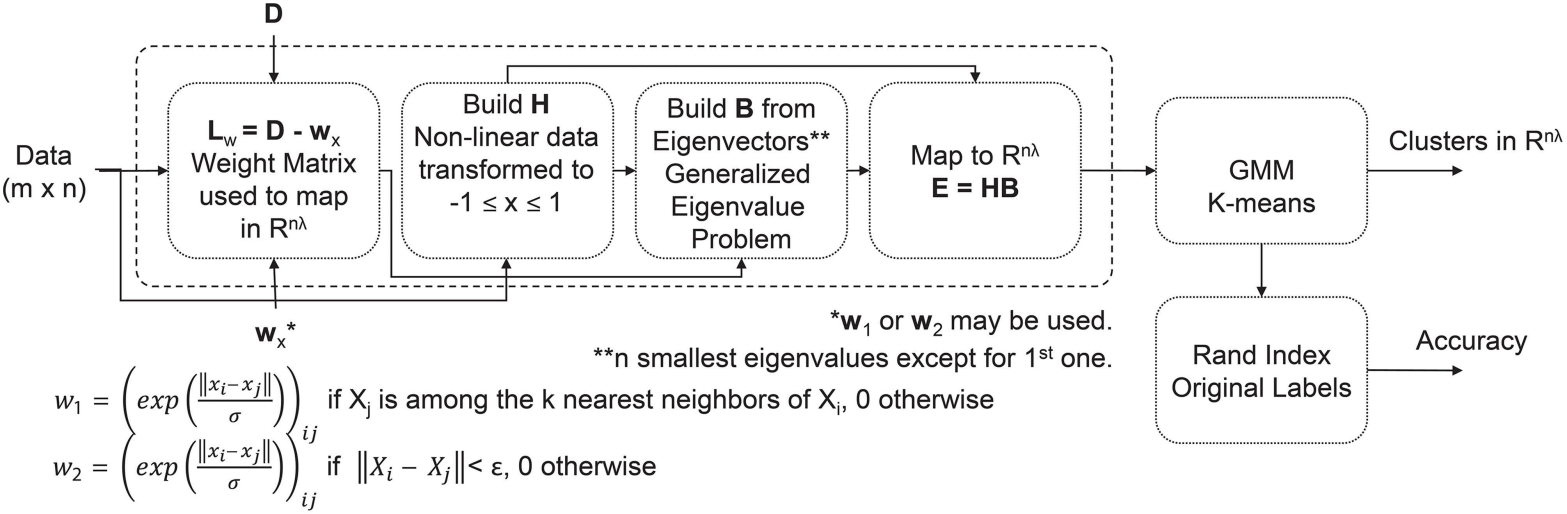
**Flow chart of clustering method for visually pleasing pieces in the exhibit**.

For the clustering analysis, we analyzed those pieces deemed visually pleasing by participants, and those pieces deemed visually complex by the image analysis. Depending on the feature domain, there could be as many as 480 features per piece per subject (e.g., time domain features: 24 features/channel ^*^ 20 channels = 480 features). Minimum Redundancy Maximum Relevancy (mRMR) is a feature selection algorithm that uses mutual information to identify features important for distinguishing between different EEG patterns, in this case associated with piece viewing (Peng et al., 2005). For our study, the 50 most important features for distinguishing between pieces were selected and used as input to an unsupervised Extreme Learning Machine (USELM) (Belkin and Niyogi, 2002; Gao et al., 2014). The USELM is used to map original features to a new space. Input data were normalized and weights additionally made orthonormal. A sigmoid function was used for the calculation of the output matrix of hidden neurons (H), generated according to ELM method (Gao et al., 2014). A Gaussian Mixture Model (GMM) and k-means method using three classes were used to cluster the output matrix from the USELM algorithm using 100 iterations to find the optimal parameters for the number of eigenvectors (nλ), number of nearest neighbors (k), and the output weight matrix between the hidden neurons and the output nodes (w). The RandIndex method, evaluating pair agreements and disagreements within proposed clusters and actual class indices, was used for accuracy reporting. A diagram of the clustering method is shown in Figure 2. The time, frequency, and wavelet domain features were used in the USELM algorithm and clustering accuracy was reported.

### Functional connectivity analysis

In order to investigate the neural networks engaged during aesthetically pleasing experiences, we performed a functional connectivity analysis implementing a frequency-domain estimator of causal interactions using the MATLAB toolbox eConnectome (He et al., 2011). The resting state network, during the baseline condition, of each subject was also analyzed.

An adaptive Directed Transfer Function (ADTF) implementing a multivariate adaptive autoregressive (MVAAR) model was used to estimate the time-varying connectivity coefficients over the frequency range of 1–50 Hz. For each time series, the MVAAR model was constructed according to the following equation (Wilke et al., 2008):

(3) $$\begin{array}{l} X\left(t\right)=\overset{p}{\underset{i=1}{\sum }}\Lambda \left(i,t\right)X\left(t-i\right)+E\left(t\right) \end{array}$$

Here, *X(t)* is the data vector over time, Λ*(i,t)* are the matrices of time-varying model coefficients, *E(t)* is multivariate independent white noise, and *p* is the model order. The time-varying coefficient matrices were established by the Kalman filter algorithm. Equation 3 can be transformed into the frequency domain, establishing the definition of the DTF function, H(f).

(4) $$\begin{array}{l} \Lambda \left(f\right)X\left(f\right)=E\left(f\right)\;where\;\Lambda \left(f\right)=\overset{p}{\underset{k=0}{\sum }}\Lambda_{k}e^{-j2\pi f△tk} \end{array}$$

(5) $$\begin{array}{l} X\left(f\right)=\Lambda^{-1}\left(f\right)E\left(f\right)=H\left(f\right)E\left(f\right) \end{array}$$

Since the time-varying model coefficients Λ*(i,t)* were characterized, the function *H(f,t)* can be obtained from the time-varying transfer matrix (Wilke et al., 2008). The elements of this matrix, Hij, represent the connection between the jth and ith elements of the system for each time point, *t*. The directional causal interaction from the jth to ith element can be described by the following equation (Wilke et al., 2008; He et al., 2011):

(6) $$\gamma^{2}\left(f,t\right)=\frac{{\left|H_{ij}\left(f,t\right)\right|}^{2}}{\sum_{m\;=\;1}^{n}{\left|H_{im}\left(f,t\right)\right|}^{2}}$$

**Figure 3:**
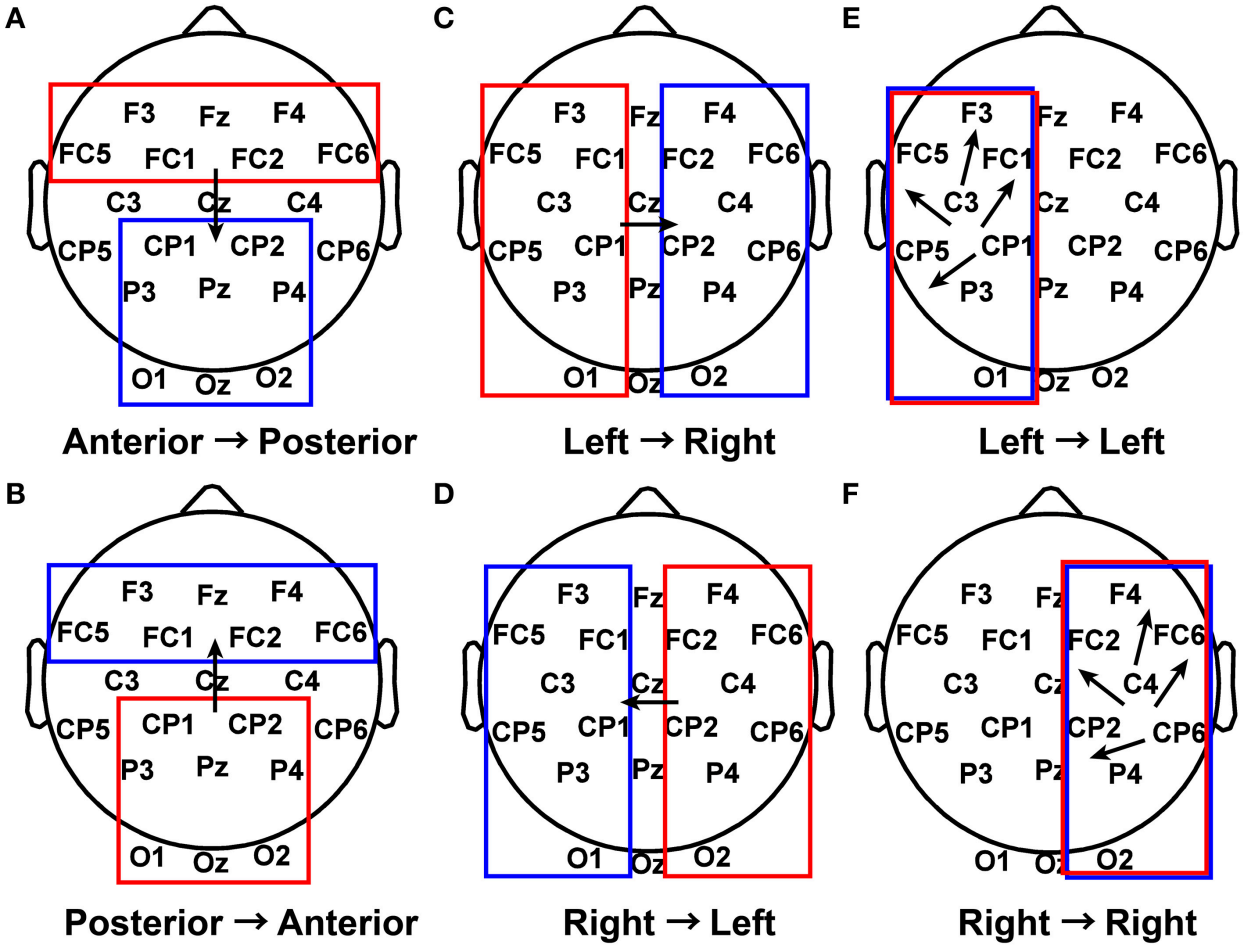
**Patterns of interest in functional connectivity analysis**. **(A)** All possible connections from electrodes in the anterior region to electrodes in the posterior region were grouped together for each subject during Piece 1 viewing and baseline. **(B)** Posterior to anterior connections. **(C)** Left to right connections. **(D)** Right to left connections. **(E)** Left to left connections. **(F)** Right to right connections.

The first 5 s of piece viewing for each subject were used as input to the ADTF algorithm. Connectivity coefficients greater than 0.3 for a specified frequency band (delta from 1 to 4 Hz, theta from 4 to 7.5 Hz, alpha from 8 to 12 Hz, beta from 15 to 25 Hz, or gamma from 30 to 50 Hz) were concatenated into a single vector for each of several defined patterns (Figure 3) for each subject during their piece viewing and baseline conditions (Fingelkurts et al., 2007). The threshold value of 0.3 was selected as a balance between selectivity for strong connections, favoring inclusion of fewer data points, and statistical power of the results, favoring inclusion of more data points. Statistical significance was assessed using the Wilcoxon *t*-test (Weiss and Rappelsberger, 2000; Fingelkurts et al., 2007).

## Results

### Subject aesthetic preferences

**Figure 4:**
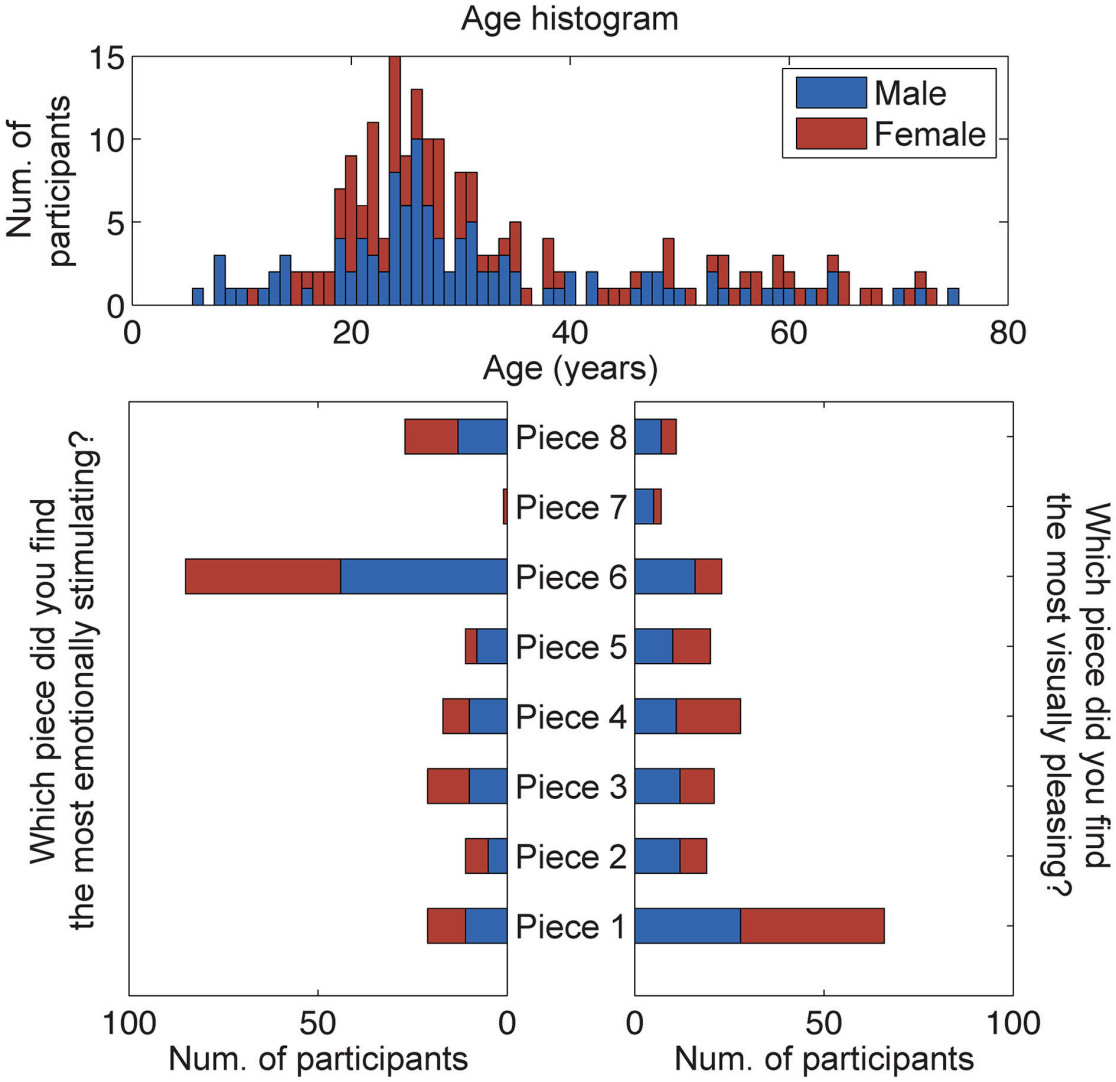
**Results of the questionnaire data (*n* = 207)**. Participants were asked to select the most emotionally stimulating and aesthetically pleasing pieces. The age and gender distributions of all participants, volunteering questionnaire data for this study, are also shown.

A total of 207 subjects provided responses to the questions: Which piece did you find the most aesthetically pleasing? Which piece did you find the most emotionally stimulating? An analysis of these responses shows that most subjects selected Piece 1 as the most aesthetically pleasing piece and Piece 6 as the most emotionally stimulating piece (Figure 4). A χ^2^-squared test of independence was performed to examine the relationship between piece preference and gender. The results were not significant, indicating that for our population, gender had no bearing on the selection of the most aesthetically pleasing piece (χ^2^ = 9.93, *N* = 207, *p* = 0.192) or the most emotionally stimulating piece (χ^2^ = 2.72, *N* = 207, *p* = 0.843).

A subset of participants that donned the reference 32-channel active EEG system was selected for further analysis of brain responses to aesthetic experiences (results from the dry EEG headsets, including signal stability and artifactual components, will be reported elsewhere). This cohort was comprised of 20 adult subjects (16 male, 4 female), and subsequent analyses of these responses shows that most subjects also selected Piece 1 as the most aesthetically pleasing piece (22% of studied subjects) and Piece 6 as the most emotionally stimulating piece (50% of studied subjects) The remaining piece preference responses were fairly well distributed between the other art pieces. The mean ages of all males and females using the BP gel headset were 26.13 ± 7.65 and 30.25 ± 12.91 years, respectively. No significant differences were found between the ages of the male and female participants (paired sample *t*-test, *p* > 0.1).

### Hierarchal clustering of features

**Figure 5:**
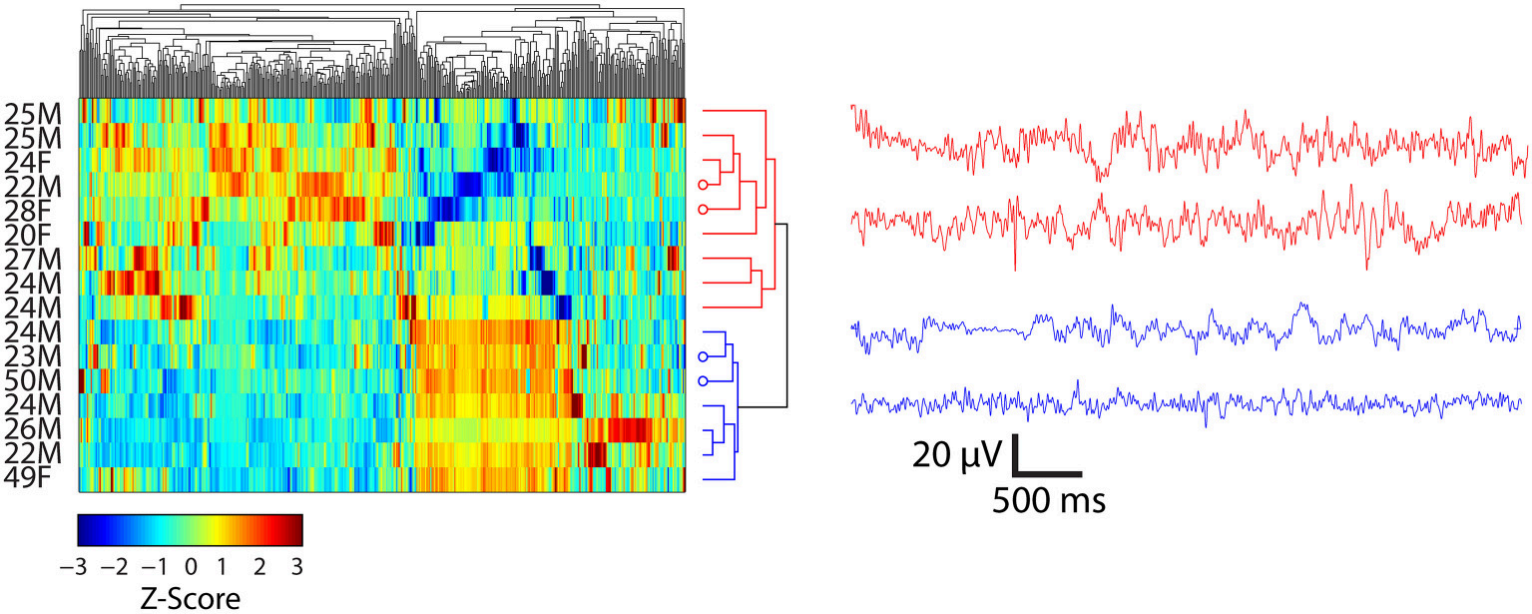
**Clustergrams (left) of standardized EEG signals and representative EEG records (right) from subjects viewing Piece 6 (*n* = 16)**. EEG traces at right are from channel FC6 for the indicated subjects. Average ± SD ages for two clusters (depicted as red and blue) of participants were 24.3 ± 2.4 and 31.1 ± 12.6 years, respectively. Open circles at the end of the dendrogram tips indicate the rows matching EEG traces at the right. Vertical ordering of EEG traces matches the vertical order of their corresponding clustergram rows.

Hierarchal clustering of the standardized time domain features (kurtosis, standard deviation, Shannon entropy, and maximum amplitude) and participants viewing Piece 1 and Piece 6 was performed. The results for participants who viewed Piece 6 (deemed by the participants to be the most emotionally stimulating) are shown in Figure 5. As a preliminary analysis of gender and age differences in the feature space, the first two groups that emerged as a result of the clustering are highlighted in red and blue on the dendrogram. The first 5 s of bandpass filtered EEG data between 0.1 and 50 Hz for two participants in each of the defined groups is shown in each plot as well. The clustering resulted in two major groups of participants depicted as red traces and blue traces. Their corresponding rows in the clustergram are indicated by the four open circles at the dendrogram tips.

The clustering results show a separation of females and males while viewing Piece 6 (Figure 5). Three out of the four females that viewed this piece were clustered together in the first group (highlighted in red). The mean age also differs by ~ 7 years, but this result is not significant (paired sample *t*-test, *p* = 0.132).

### Image analysis

**Figure 6:**
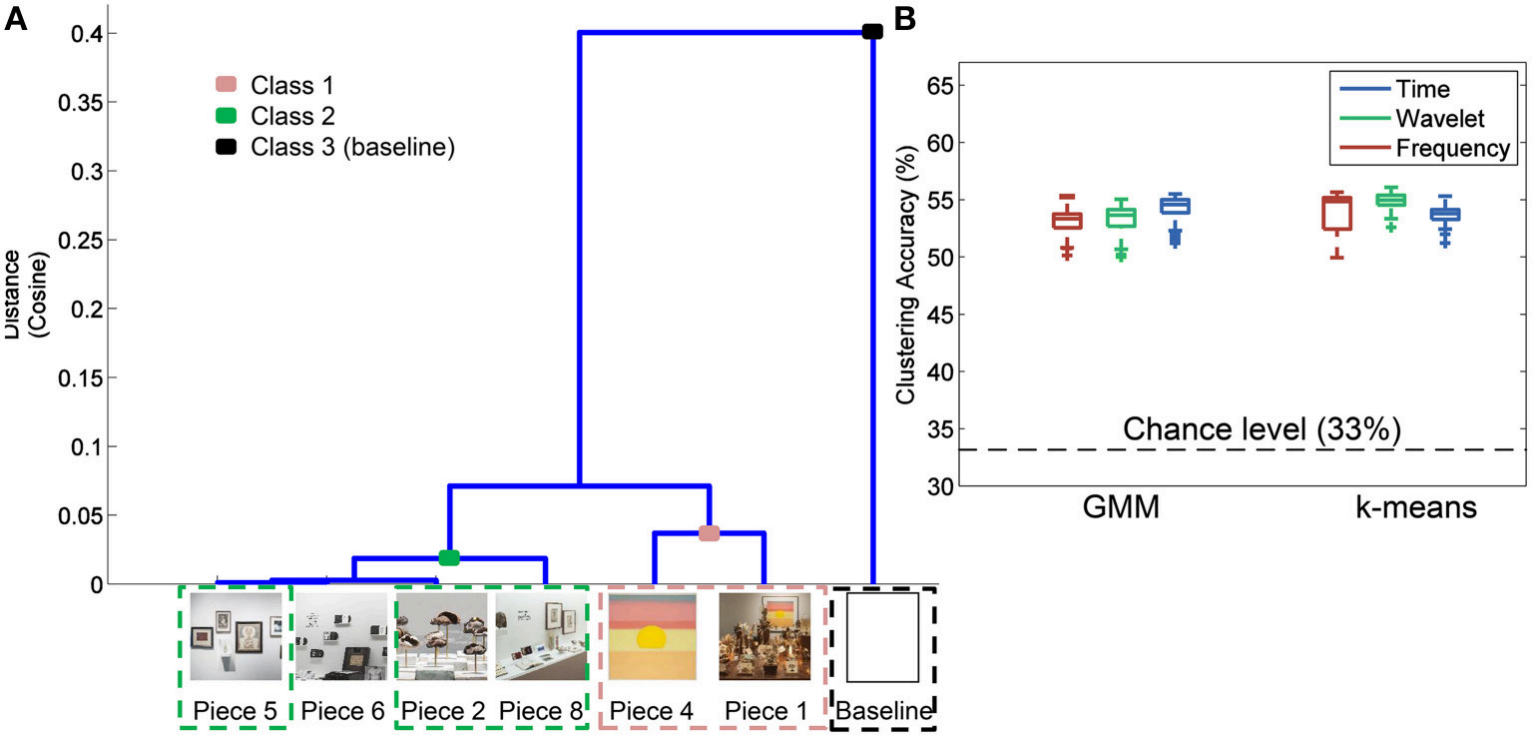
**Image analysis and clustering accuracy results**. **(A)** Hierarchal clustering of image-based features to determine those pieces in the exhibit that shared certain visual aspects. Piece 3 was not included in the analysis since a complete image of the piece was not available. **(B)** Clustering accuracy results using the groupings identified in **(A)** for three different feature domains and two clustering methods.

Figure 6A shows the results of the hierarchal clustering of artwork images using the cosine as the distance metric. An image of a white wall was also included in the image analysis to represent the visual input during baseline data collection. Three major groupings emerged as a result of this image analysis: Class 1 (Pieces 1, 4); Class 2 (Pieces 8, 2, 6, and 5); and Class 3 (baseline). These groupings were also reflected in the questionnaire data rating the subjects' preference for the most aesthetically pleasing piece (see Figure 4). Subjects who visited the pieces listed above were included in the analysis to correlate the human brain responses evoked during the aesthetically pleasing viewing experience with the different pieces. Clustering analysis was performed with three classes, as delineated above, with the exception that piece 6 was eliminated, since it was determined to be the most emotionally stimulating piece, and also had several artifacts from the audio content associated with the piece. All other pieces from the exhibit had purely visual content.

### Feature selection and clustering

The results of the three-class clustering analysis using GMM and k-means are shown in Figure 6B. For each feature domain and clustering method, 100 iterations were performed to obtain a distribution of clustering accuracies, reflected as box plots in Figure 6B. Average accuracy of 55% was obtained with the time domain features and a GMM clustering method. This was significantly greater than chance-level at 33% (paired sample *t*-test, *p* < 0.01).

**Figure 7:**
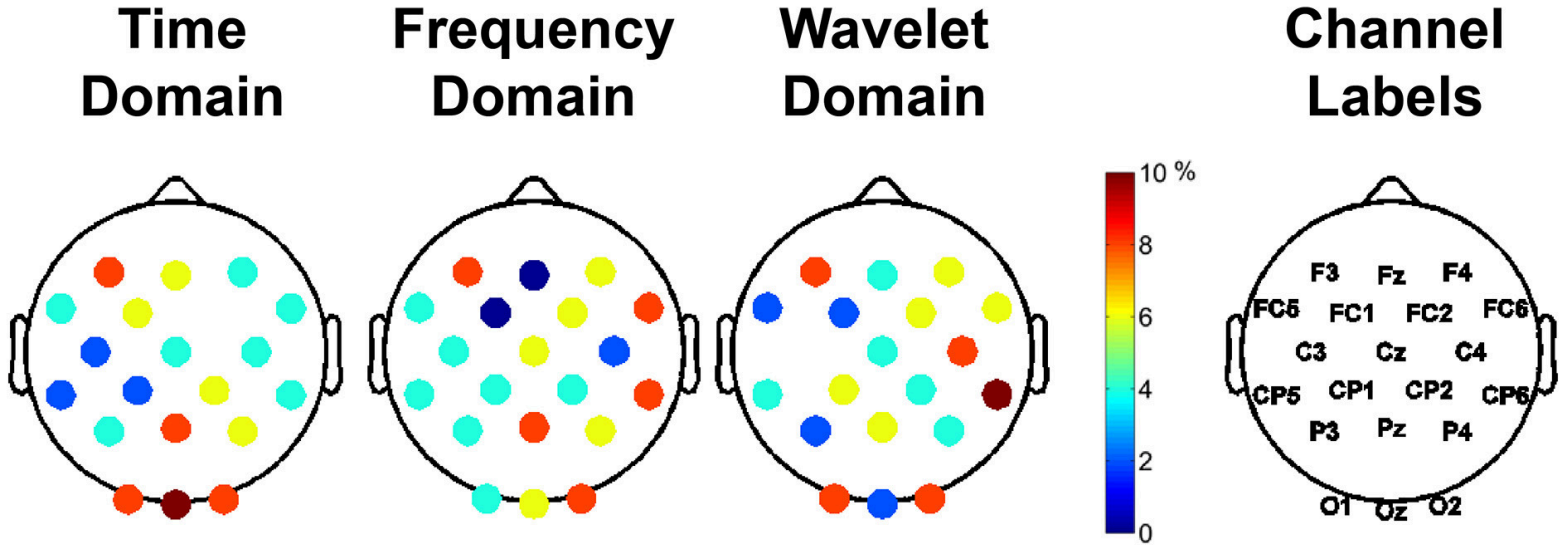
**Important channels for distinguishing between different aesthetically evoking visual art pieces according to the feature selection algorithm, mRMR**. Each column represents the different feature domains used to cluster the data. The different colors represent the percentage of use of that particular channel in the selected most important 50 features for clustering. The rightmost column shows the labels of the channels.

The most important 50 features for clustering the data were computed for each feature domain using the mRMR feature selection algorithm (Peng et al., 2005). The representation of each channel in the top 50 features is shown in Figure 7, with the color bar reflecting the percentage of the 50 features that a given channel represented. For example, 25% of the features used for clustering in the time domain came from channels O1, Oz, and O2. For each feature domain, those channels in the posterior and anterior regions were more often selected as containing the important features.

**Figure 8:**
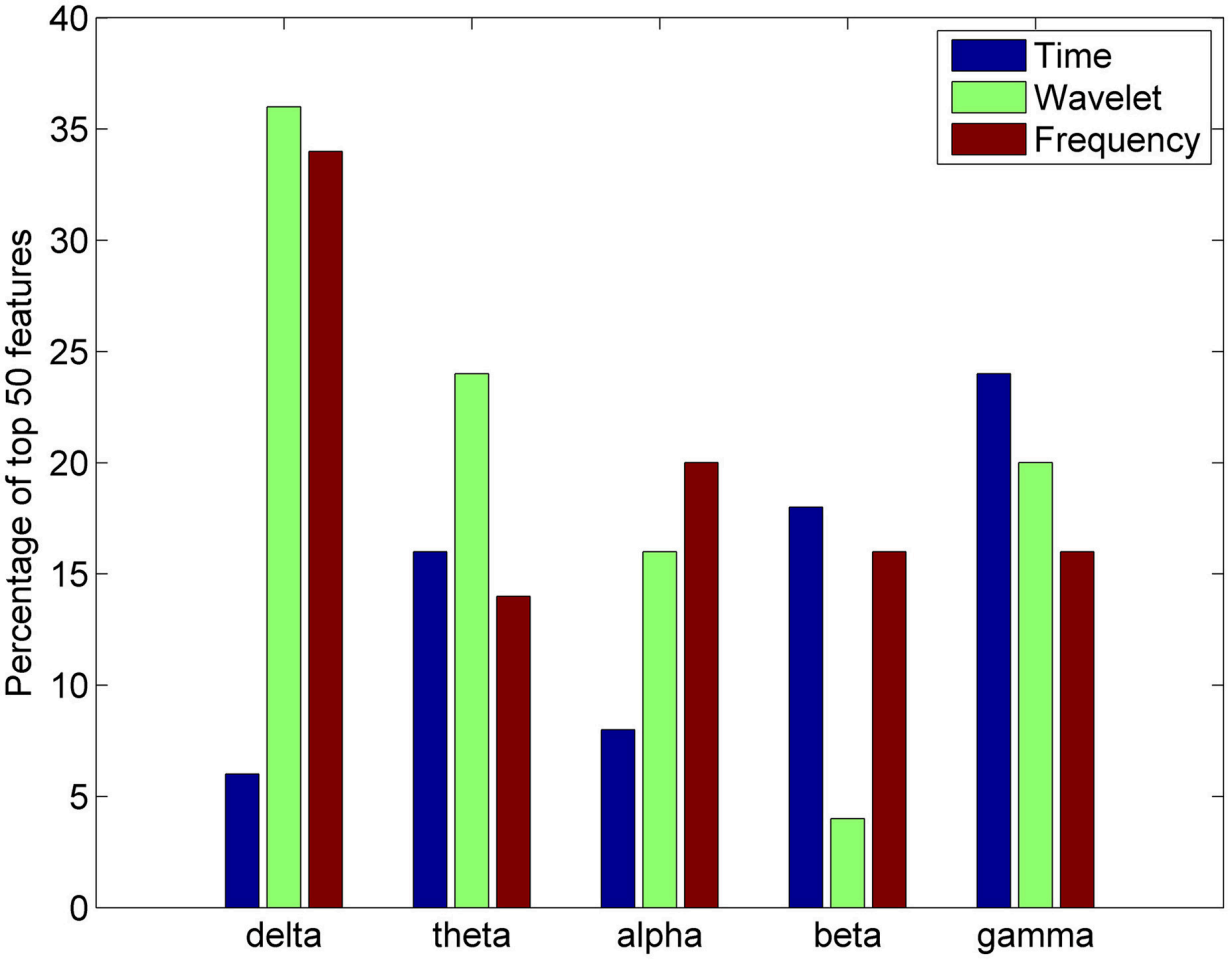
**Percentage of top 50 features in the time, wavelet, and frequency feature domains from each frequency band for the all studied subjects (*n* = 20)**.

For each feature domain, various parameters were calculated in five different frequency bands: delta (1–4 Hz), theta (4–7.5 Hz), alpha (8–12 Hz), beta (15–25 Hz), and gamma (30–50 Hz). An analysis was also done to ascertain the most important frequency band in differentiating between complex artistic stimuli and baseline data. The percentage of the top 50 features selected for clustering from each of the five frequency bands was calculated for the time, frequency, and wavelet feature domains for all subjects. Results indicate that the delta and gamma bands were more often selected as top important features across all feature domains (Figure 8).

### Functional connectivity

**Figure 9:**
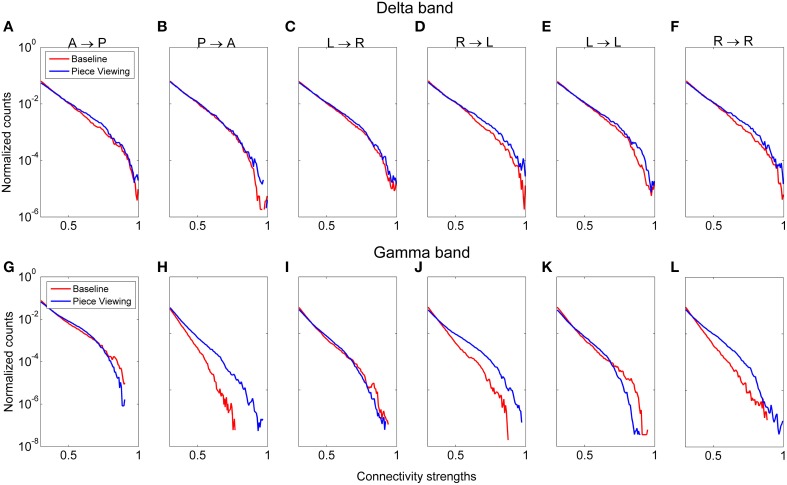
**Distribution of connectivity coefficients for predefined patterns (cf. Figure 3) using EEG data from viewing Piece 1 (blue line) and the baseline data (red line)**. Distributions are shown for the delta band **(A–F)** and gamma band **(G–L)**. Distributions are normalized to the number of counts in each connectivity strength bin. All comparisons between piece viewing and baseline data for each pattern are statistically significant (Wilcoxon test, *p* < 0.05).

Functional connectivity analysis was performed to determine the broad active neural networks during piece viewing and to determine any statistical differences in the connection strengths for predefined patterns (Figure 3) between piece viewing and resting baseline conditions. The connectivity coefficients from all subjects viewing Piece 1 (*n* = 16) over the entire 5 s time period for a specified frequency band were tested against those same subjects' baseline connectivity coefficients over a 5 s time period for the same frequency band using the Wilcoxon test. The specified frequency bands used were as follows: delta (1–4 Hz), theta (4–7.5 Hz), alpha (8–12 Hz), beta (15–25 Hz), and gamma (30–50 Hz). For all patterns, the connectivity coefficients from subjects viewing Piece 1 were statistically greater than the connectivity coefficients from the subjects' baseline (Wilcoxon test, *p* < 0.05). Figure 9 shows the distributions of the connectivity coefficients for all subjects over two frequency bands (delta and gamma bands) that were often selected as top important features across all feature domains. Distribution results for the theta, alpha, and beta frequency bands followed a similar trend (See Supplemental Videos 1, 2 for functional connectivity changes over time in the alpha band for one subject during baseline and piece viewing).

Pattern D (right → left hemisphere) showed the most pronounced difference between baseline and piece viewing for both delta and gamma frequency bands. For Pattern B (posterior → anterior), the difference between baseline and piece viewing in the gamma band was more pronounced than for the delta band. These differences usually occurred at higher connectivity weights.

**Figure 10:**
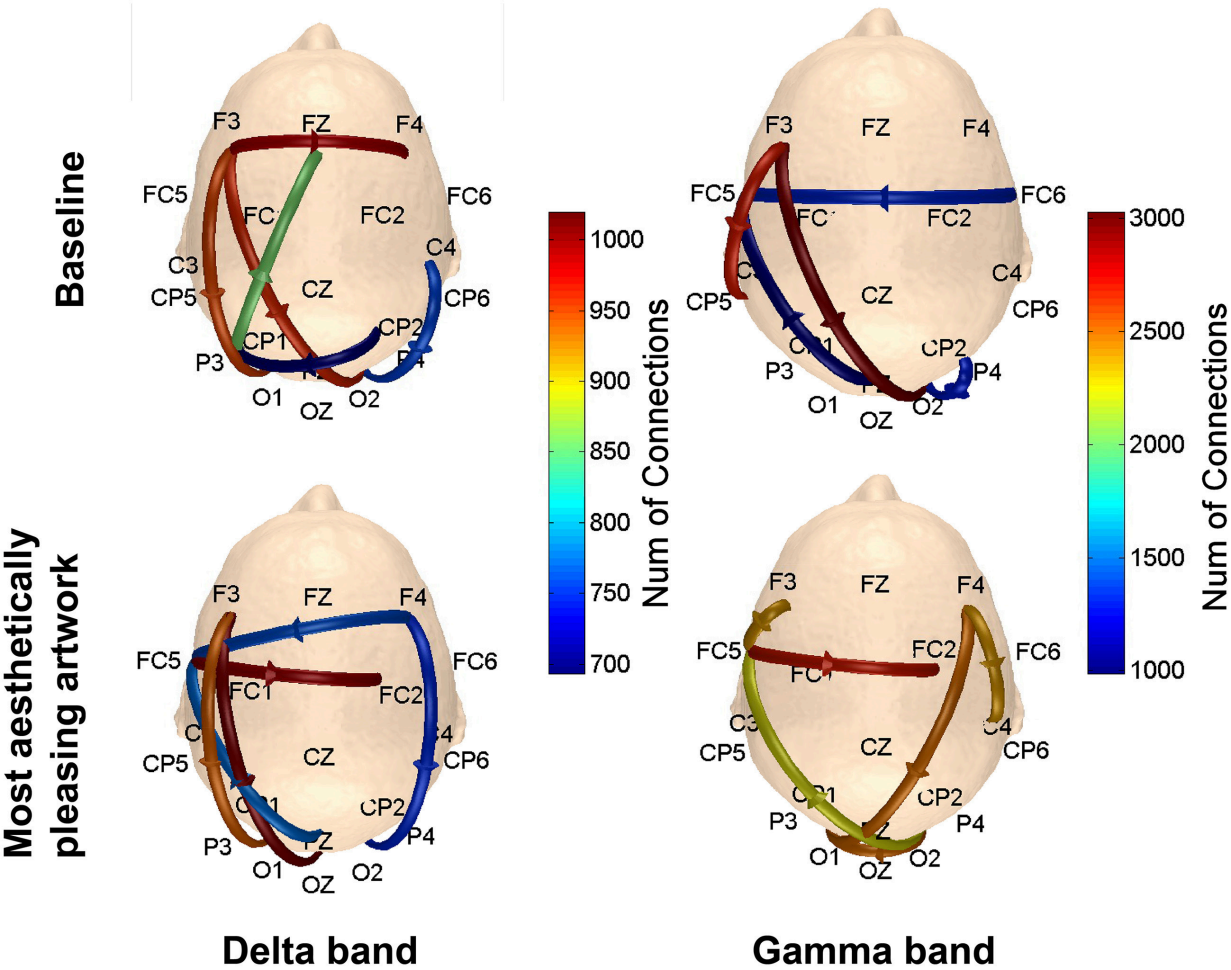
**Scalp maps showing the number of connections between functionally-related pairs of electrodes**. The channel that yielded the most connections (averaged over subjects) within each defined pattern was plotted on the scalp map. Results are shown for the delta and gamma bands.

To determine the connections most prominent within each of the defined patterns in Figure 3, the number of connections with a connectivity coefficient greater than 0.3 over the entire 5 s viewing period were counted for every possible combination of channels in a defined pattern, and averaged over subjects. The results are shown in Figure 10. In the delta and gamma frequency bands, there was more activity from the visual cortex to the frontal lobe, followed by additional neural activity in the frontal lobe for subjects viewing art compared to baseline data. The right visual cortex served as the starting point for communication to the right frontal region for the delta band, and to the left fronto-parietal region covered by posterior and anterior electrodes for the gamma band.

The data in Figure 9 used all connections for a given pattern throughout the 5 s time period to provide a general and global statistical measure of the difference between piece viewing and baseline data. In order to get a better understanding of how the connectivity of the defined patterns for each subject changed as a function of time, the 5 s data was also divided into shorter 1-s segments for each subject.

**Figure 11:**
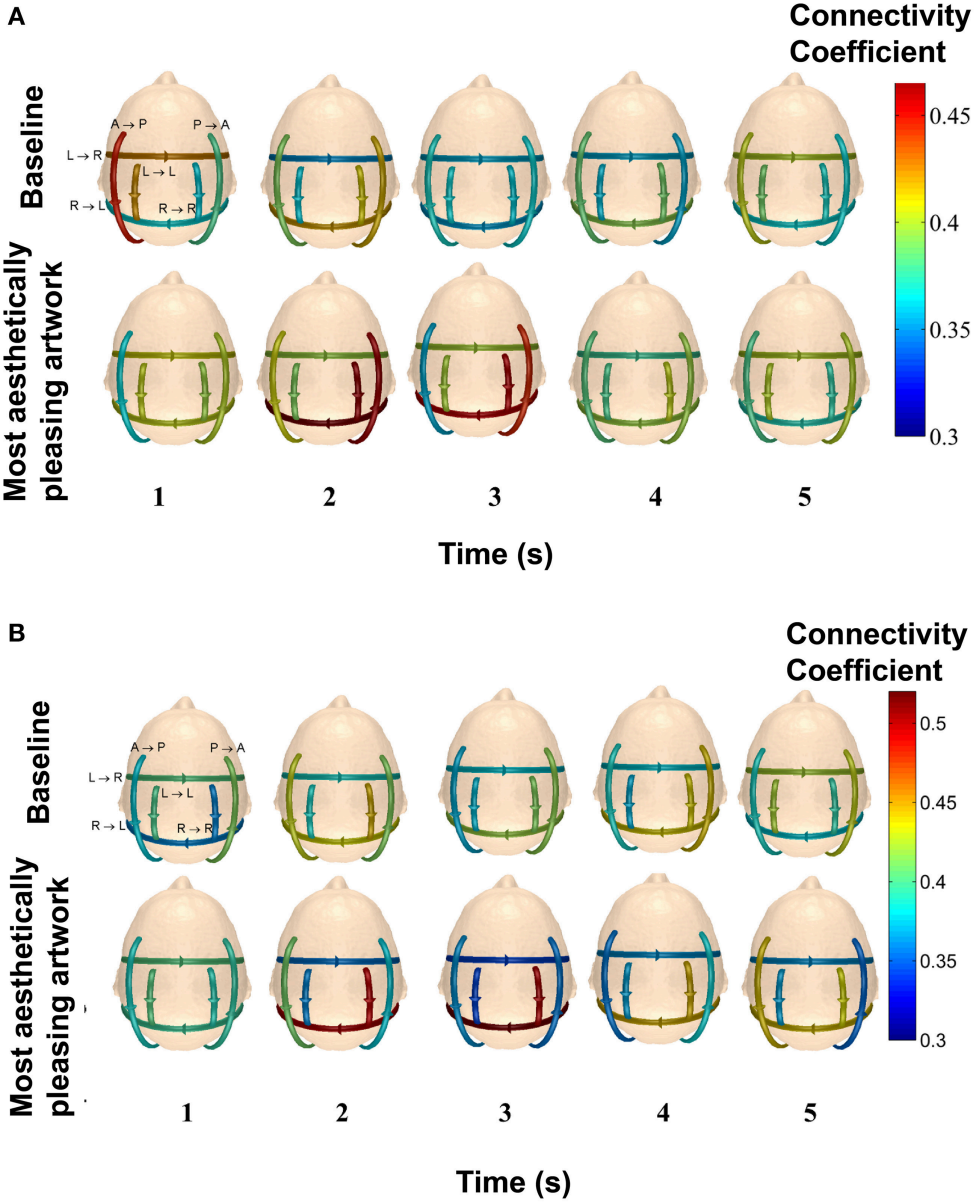
**Average connectivity coefficients for the defined patterns over 1-s time intervals in the delta band for two participants**. The top row of scalp maps represents the baseline data. The bottom row of scalp maps represents the Piece 1 viewing data. The patterns that each line arrow represents are shown in the top left scalp maps. **(A)** The subject is a 49 y/o female. **(B)** The subject is 27 y/o/ male.

Figure 11 shows the average connectivity coefficient strength for five, 1-s time intervals for two subjects (49 y/o female and 27 y/o/ male) in the delta band. Each arrow on the scalp map represents a specific pattern, with the represented pattern next to each arrow on the top left scalp map. From Figure 11A, it can be seen that this particular subject had very high connectivity in Pattern B (posterior → anterior), Pattern D (right → left hemisphere), and Pattern F (right → right hemisphere) within the first 3 s of piece viewing compared to the baseline data. In Figure 11B, the stronger connection strengths for Pattern D (right → left hemisphere) and Pattern F (right → right hemisphere) occurred over the second and third seconds of piece viewing. The baseline connectivity strengths for all patterns are similar for both subjects.

**Figure 12:**
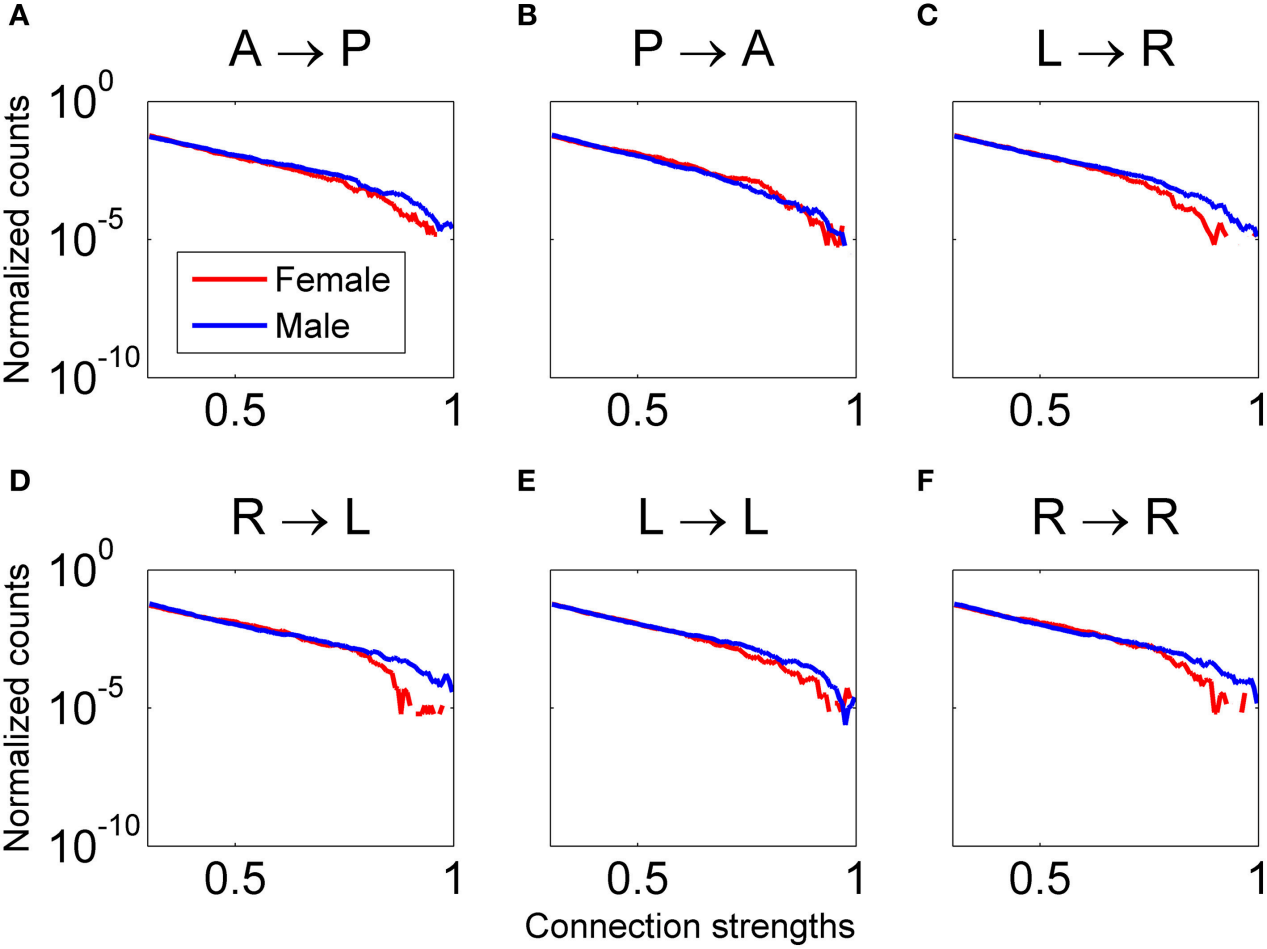
**Distribution of connectivity coefficients for predefined patterns using data from all female (red line) and male (blue line) subjects viewing Piece 1**. Distributions are shown for the delta band. Distributions are normalized to the number of counts in each connectivity strength bin. All comparisons between male and female for each pattern are statistically significant (Wilcoxon test, *p* < 0.05). In plots **(A, C–F)**, males have significantly higher connectivity coefficients. In plot **(B)**, females have significantly higher connectivity coefficients.

A comparison between all the males and females that viewed Piece 1 was also done to determine if any differences in functional connectivity during perception of complex objects occurs. The results shown in Figure 12 indicate that for our sample population, males had significantly higher connectivity strengths for all defined patterns, except for Pattern B (posterior → anterior), in the delta frequency band (Wilcoxon *t*-test, *p* < 0.01). In the gamma band, the males had significantly higher connectivity strengths than females in all defined patterns.

**Figure 13:**
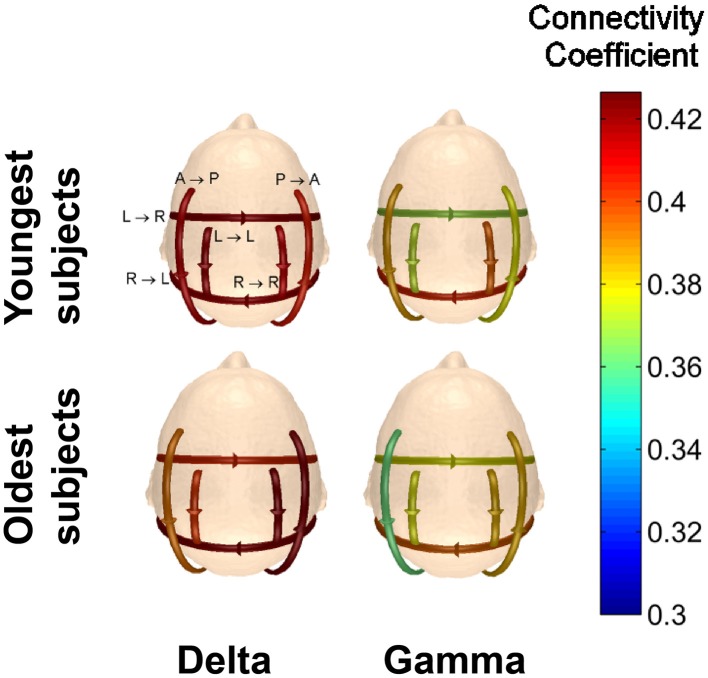
**Comparison of the average connectivity coefficients for the two youngest subjects and the two oldest subjects viewing Piece 1**. The average coefficients were calculated for each frequency band.

A comparison of the connectivity strengths for the two youngest subjects (22 y/o male and 20 y/o female) and the two oldest subjects (50 y/o male and 49 y/o female) while viewing Piece 1 was also done to determine if age yields any significant differences in the visual perception of complex objects. The average connectivity strength for each of the defined patterns was calculated and plotted for the five different frequency bands. The results in Figure 13 indicate that for our small sample size, we can see differences in the strength of the connections as a function of age.

For every frequency band, the youngest subjects had higher average connectivity coefficients for the anterior → posterior pattern, while the oldest subjects had higher average connectivity coefficients for the posterior → anterior pattern. For the pattern of connectivity from left → right, the connectivity coefficients for the oldest subjects is lower than those for the youngest subjects in the delta band. In the higher frequency bands, however, the connectivity strengths of the oldest subjects are significantly higher than the youngest subjects for the connectivity pattern from left → right (Wilcoxon *t*-test, *p* < 0.01).

## Discussion

In the present study, neuroimaging, aesthetic and emotional preference data from over 400 subjects were collected over 3 months from people of various ages, genders, educational levels, careers, health statuses, and neurological backgrounds using various types of dry and gel-based EEG systems. The underlying goal of this study was to determine the feasibility of acquiring useful data from freely moving participants in a real, complex setting such as a museum, and findings from the present work indicate that our proposed approach is indeed feasible. Using the artwork from conceptual artist Dario Robleto as the common stimulus for participants, we were able to quantify the aesthetic experience through the analysis of brain differences that arise between participants during an aesthetic judgment, and through functional network connectivity. Here, we report analysis of brain activity from a small cohort of participants who used a gel-based reference active EEG system with typically higher signal-to-noise ratio and less susceptible to motion artifacts than dry EEG systems. The main conclusions that can be drawn from this work revolve around differences between various subgroups within the subject population, and the areas of the brain implicated in the perception of aesthetically pleasing artwork. The following sections discuss these conclusions further.

### Regional brain connectivity: Initial evaluation of age and gender differences

Mobile EEG shows promise for non-invasive decoding of user intent (Kilicarslan et al., 2013; Bulea et al., 2014; Hernandez et al., 2014; López-Larraz et al., 2014; Loza et al., 2014; Agashe et al., 2015) and emotional state (Petrantonakis and Hadjileontiadis, 2011; Hadjidimitriou and Hadjileontiadis, 2012; Kim et al., 2013). In addition, quantitative EEG metrics may play increasingly important diagnostic roles (Mormann et al., 2000; Adeli et al., 2007; Ahmadlou and Adeli, 2010). For all these reasons, it is critical to understand the variability in the quantitative EEG signal across different populations of individuals in real complex settings (Cantillo-Negrete et al., 2013; McIntosh et al., 2014; Tello et al., 2014). This initial analysis highlights consistent data from three promising directions in the study of the neural basis of aesthetic experiences from brain signals of freely behaving participants acquired in an unconstrained environment.

First, the clustergram analysis sorted subjects into two major groups according to the most important features as determined by mRMR. These groups did not differ in mean age; however, the only two participants not in their 20s (one 49yo male and one 50yo female) clustered to the same group. The other three females clustered to the other group. This suggests a probable age effect to investigate in future studies.

Analysis of the regional brain activity in all relevant frequency bands while participants viewed a widely-considered highly aesthetically pleasing conceptual art piece (Piece 1, voted the most aesthetically pleasing by approximately 22% of the 20 study participants) showed that male connectivity strengths were significantly higher in all of the defined regional patterns except for the pattern linking posterior and anterior brain regions in the 1–4 Hz range. For this pattern, shown in Figure 12B, the four female participants in this study generated significantly higher connectivity strengths compared to the 12 male participants, which is associated with visual processing and high-order decision-making and planning computations.

Several studies have examined active brain networks during certain tasks and during baseline conditions for the developing brain (children < 20 y/o; Micheloyannis et al., 2009; Boersma et al., 2011), but comparatively fewer investigations have focused on functional connectivity in the aging brain (Tomasi and Volkow, 2012). One study reported a decrease in the relative power contribution in the left temporal-occipital and the right inferior frontal brain regions in the alpha band for subjects between the ages of 50 and 89 years when compared to subjects between the ages of 20 and 29 years (Shinosaki et al., 2003). Results in the current study indicate that different patterns produce stronger connection coefficients depending on the age of the subject. On average, the older participants had stronger connections between the posterior and anterior regions of the brain, as well as from the right to left hemisphere in the studied frequency bands (Figure 13). Results obtained in the current study show a decrease in the average recurrent connectivity strength in the left → left hemisphere pattern, which is consistent with reports in the literature (Shinosaki et al., 2003). Future work could include the evaluation of functional connectivity of smaller regions as well as frequency-specific source analysis to get a better understanding of the local networks active during aesthetic judgments and experiences (Micheloyannis et al., 2009).

Given the limited number of participants analyzed in this initial work (only 20 subjects donned the active gel-based system, which required time-consuming setup and left participants with gel residues), definitive conclusions about how people of different age and gender perceive artwork cannot be made. Future studies would require the collection of data from more subjects to increase the statistical power so that any differences between these groups can emerge.

### Role of visual and frontal regions in aesthetic judgments

After initial segmentation of data, the subjects were grouped into three categories of piece viewing based on the image analysis: complex, moderate, and baseline. The feature selection based on minimum redundancy maximum relevance (mRMR) selected the channels spanning the visual (O1, OZ, O2, PZ) and frontal (FC5, F3, and F4) scalp areas as being the most important for clustering (Figure 7). Since the artistic stimulus was visual, it is not surprising to find important features in these brain regions. The visual cortex is responsible for processing visual input, while emotional expression and judgments are mainly localized to frontal areas (Moll et al., 2002). More or less activity in the frontal region could indicate a propensity to approach or engage a stimulus, or withdraw from it (Demaree et al., 2005; Babiloni et al., 2013). Further analysis of the important features in time, frequency and wavelet domains for clustering the EEG data revealed the significance of the delta and gamma bands, which are related to long-range and short-range cortico-cortical communication, respectively (Munk and Neuenschwander, 2000).

These findings from the mRMR analysis were corroborated with the functional connectivity results showing strong links between occipital and frontal brain regions during piece viewing in the delta and gamma frequency bands. More specifically, electrodes F3 and FC5 showed an increase in the number of connections with electrodes O1, OZ, and PZ in the delta band. Numerous connections between posterior (parietal) and anterior (frontal) regions during the first 5 s of piece viewing in the delta band were also seen in comparison to the baseline connectivity. Delta band activity has been shown to reflect sustained attention (Kirmizi-Alsan et al., 2006) as well as a linkage between parietal and frontal cortical circuits during decision making (Nácher et al., 2013). The important brain regions identified through the mRMR analysis are consistent with studies reporting activation of anterior occipital and anterior parietal regions during both visually pleasing and not visually pleasing stimuli (Cela-Conde et al., 2013).

Functional connectivity in the gamma band increased substantially during piece viewing compared to baseline, and also compared to connectivity in the delta band. The number of functional connections during piece viewing in the gamma band ranged between 2300 and 3000, whereas the number of delta band connections during piece viewing ranged from 700 to 1000. This finding is consistent with previous reports concluding that gamma band oscillations increase after attention to visual stimulation (Fries et al., 2001; Bauer et al., 2014). Additional studies have also shown that synchrony in gamma band oscillations increases during perception of visual art (Bhattacharya and Petsche, 2002). It has been noted that gamma-oscillatory responses can synchronize with millisecond precision over long distances and are mediated by the reciprocal corticocortical connectivity (Munk and Neuenschwander, 2000). Moreover, it is likely that during high states of functional cortical activation, the functionally-relevant frequencies of the EEG may shift from lower frequencies in the delta range to higher frequencies in the gamma-range, and they may indicate different degrees of temporal precision with which large neuronal populations interact during piece viewing in this study.

Interestingly, relative to baseline, the overlap between prominently connected regions in the delta and gamma bands was more evident during piece viewing. For both frequency bands, the normalized histograms of connectivity strengths in Figure 9 showed that the patterns linking the posterior → anterior regions, right → left hemispheres, and within the right hemisphere exhibited significantly higher connection strengths during piece viewing. This supports the notion of interdependency of these two frequency bands in the emotional and visual processing of complex artwork as noted above. It has been shown that slow wave oscillations originate from deeper, subcortical structures while faster oscillations stem from cortical structures (Michel et al., 1992; Robinson, 1999; Luo et al., 2013), and that these various frequencies synchronize in spatially distinct patterns (Buzsáki and Silva, 2012; Civillico and Contreras, 2012). Previous studies have shown cross-frequency coupling between the gamma and theta oscillations in the hippocampus during a memory task (Belluscio et al., 2012). Preliminary evidence for such a coupling in an aesthetic viewing context is presented here, but further investigation is needed to definitely demonstrate a functional relationship between frequency bands.

Finally, the combined activity of delta and gamma band oscillations over occipital and frontal brain regions resulted in features in the time, wavelet, and frequency domains that could be used with a Gaussian Mixture Model (GMM) to cluster subjects viewing artwork with varying complexities. The best model was able to predict the visual complexity of the viewed artwork with 55% accuracy, which was significantly greater than the chance level of 33% for our three complexity classes. While this result shows the feasibility of isolating useful information from freely behaving subjects, more research needs to be done to increase the classification accuracy of the extracted information. Future studies could explore the use of shorter epochs to increase the specificity of the feature results within and across subjects. However, more detailed, independent measures of location and behavior during piece viewing would need to be incorporated into the experimental set-up to determine time-resolved correlations of behavior with brain activity.

### Limitations and future work

While experimental protocols like the one employed here allow subjects to move about freely and explore the exhibit at their leisure, they do present complications for data analysis when correlating specific events (i.e., piece viewing) with brain data (Gramann et al., 2014). In our case, it is difficult to know exactly when/where a participant was fixated on a piece without eye tracking tools. This problem could be somewhat mitigated by using the data from the functional connectivity analysis to determine a time period over which the connectivity reflects the activity that should be present during a task (i.e., visual perception) compared to baseline data. For example, connectivity is expected to increase between electrodes covering the visual cortex and the frontal cortex during a visual task (Bradley et al., 2007; Cocchi et al., 2011). Looking at Figure 11A, the first 2 s of data have high connectivity strengths between those two regions of interest during piece viewing for this participant, and could be isolated from the entire 5 s period for use in clustering analyses.

Building on the data already collected, the functional connectivity can also be assessed in participants viewing other pieces of art to determine how image complexity and specific image features alter the engaged networks. Studies have shown that the contrast of a visual stimulus can alter both the strength and proximity of network connections (Nauhaus et al., 2009). This creates the possibility of investigating functional connectivity as a function of image features, such as those employed here.

The preliminary functional connectivity results reported here assumed very broad regions of interest. In future analyses, those regions of interest could be reduced to obtain a better understanding of the exact regions of activation. The analysis reported in Figure 10 indicates a start in this direction, as it evaluates of the maximum number of connections within each defined pattern (A → P, R → L, etc.). To achieve a more thorough connectivity analysis, the distance of a connection could also be taken into account (Fingelkurts et al., 2007). For example, high connectivity coefficients between signals might reflect different underlying processes depending on the spatial distance between the electrodes that recorded them. Source analysis of frequency-relevant bands (e.g., delta and gamma) could provide detailed spatial and temporal sources of such activations from neural networks in “action and context.”

### Conflict of interest statement

The authors declare that the research was conducted in the absence of any commercial or financial relationships that could be construed as a potential conflict of interest.

## Supplementary material

Video 1 **Functional connectivity during baseline data collection**. Connections were assessed every 1 ms, and data shown here span five seconds of baseline collection.

Video 2 **Functional connectivity during piece viewing**. Connections were assessed every 1 ms, and data shown here span five seconds of active piece viewing.

The questionnaire given to study participants after viewing Dario Robleto's exhbit “The Boundary of Life is Quietly Crossed” is given. An image of a study participant viewing the exhibit with the Brain Products actiCAP EEG system is also shown.
