# Corrigendum: Your Brain on Art: Emergent Cortical Dynamics During Aesthetic Experiences

**DOI:** 10.3389/fnhum.2015.00684

**Published:** 2015-12-16

**Authors:** Kimberly L. Kontson

**Affiliations:** Office of Science and Engineering Laboratories, Division of Biomedical Physics, Center for Devices and Radiological Health, U.S. Food and Drug AdministrationSilver Spring, MD, USA

**Keywords:** EEG, machine learning, functional connectivity (FC), aesthetics, freely moving

In Table 1, the descriptions for Piece 6 and Piece 7 should read as follows:

*Piece 6: The Pulse Armed With a Pen (An Unknown History of the Human Heartbeat), 2013-2014. Custom-cut five-inch vinyl records, audio recordings, archival digital prints (record sleeves and linear notes), prints of three centuries of various human pulse and heartbeat tracings*.

*Piece 7: Max Ernst, Undulating Earthquake (Tremblement de terre ondulatoire), 1928. Oil on canvas mounted on masonite. The Menil Collection, Houston*.

## Conflict of interest statement

The author declares that the research was conducted in the absence of any commercial or financial relationships that could be construed as a potential conflict of interest.

