# Supplementary material for: Your Brain on Art: Emergent Cortical Dynamics During Aesthetic Experiences
Source: Front Hum Neurosci. 2015 Nov 18;9:626. doi: 10.3389/fnhum.2015.00626 (PMC4649259; doi:10.3389/fnhum.2015.00626)
Supplement: Supplementary file 3 [file DataSheet1.DOC]

***Supplementary Material***

**‘Your Brain on Art’: Emergent cortical dynamics during aesthetic experiences**

**Kimberly L. Kontson†*^1,2^, Murad Megjhani*^2^, Justin Brantley^2^, Jesus G. Cruz-Garza^2^, Sho Nakagome^2^, Dario Robleto^3,4^, Michelle White^4^, Eugene Civillico^1^, Jose L. Contreras-Vidal^2^**

^1^Division of Biomedical Physics, Office of Science and Engineering Laboratories, Center for Devices and Radiological Health, U.S. Food and Drug Administration, Silver Spring, MD, USA

^2^Laboratory for Non-Invasive Brain Machine Interfaces, Department of Electrical and Computer Engineering, University of Houston, Houston, TX, USA

^3^Philosopher/Artist of American conceptual art, Houston, TX, USA

^4^The Menil Collection, Houston, TX, USA

*Contributed equally to this work

**† Correspondence:** Kimberly L. Kontson, Division of Biomedical Physics, Office of Science and Engineering Laboratories, Center for Devices and Radiological Health, U.S. Food and Drug Administration, 10903 New Hampshire Ave, Silver Spring, MD 20933, USA

1. **Supplementary Figures**

**
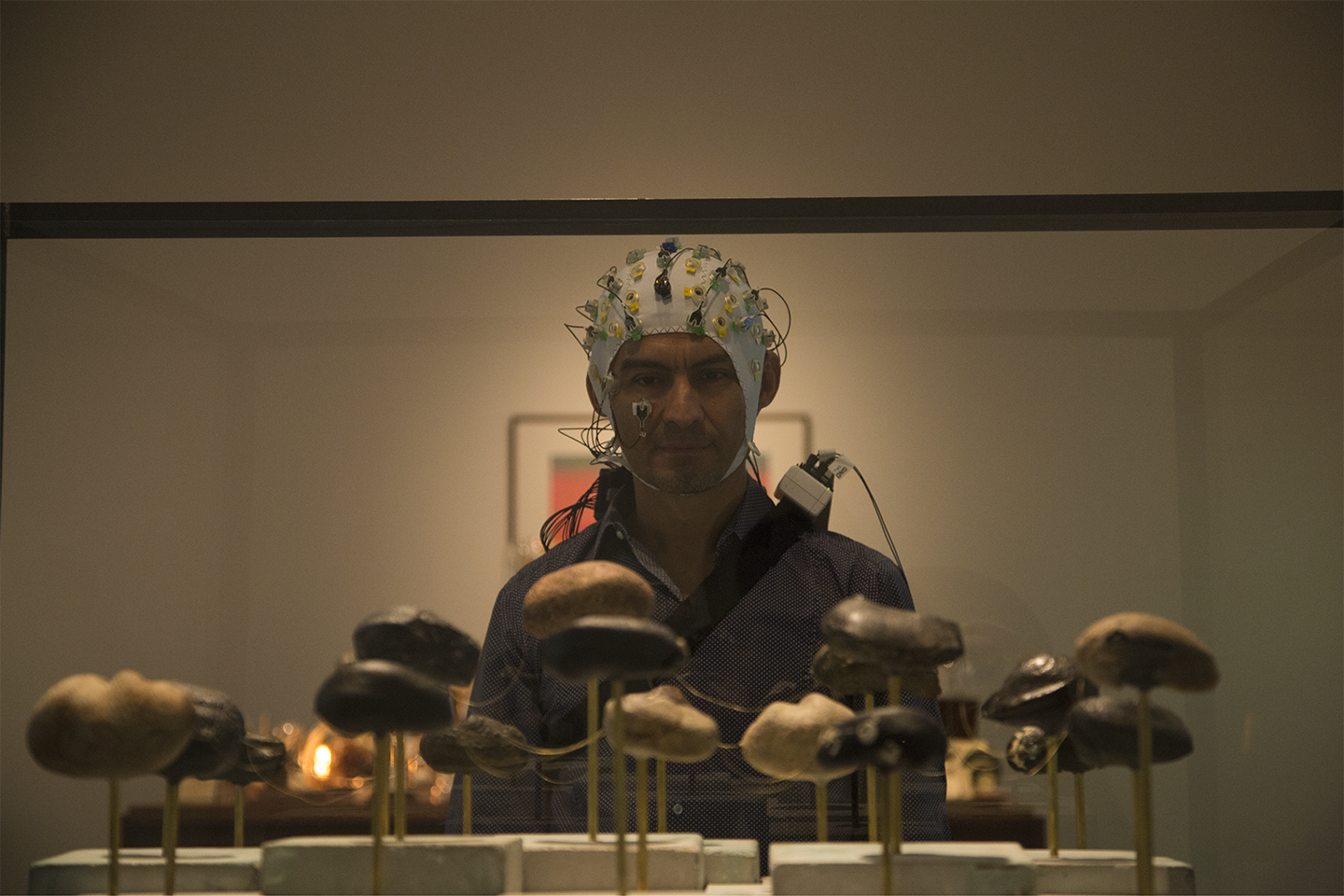
**

**Supplementary Figure 1.** An image of a study participant viewing the exhibit with the Brain Products actiCAP EEG system.

1. **Supplementary Data**

The following questionnaire was given to participants after viewing Dario Robleto’s exhibit titled “The Boundary of Life is Quietly Crossed”.

Participant Questionnaire Subject ID:__________

Please provide the following information. If the participant is a minor/child, a guardian should fill out the questionnaire.

**Age:** _____________

**Gender:** ____________

**Race (circle one):**

American Indian/Alaska Native Asian Black/African American

Native Hawaiian White

**Ethnicity (circle one):** Hispanic Non-Hispanic

Please answer the following questions to the best of your ability.

**1.** **Have you consumed any of the following within the last four hours?**

a. Caffeine Yes No

b. Alcohol Yes No

c. Recreational Drugs Yes No

**2.** **Please list any medications that you currently use on a daily basis.**

________________________________________________________________________________________

**3.** **Do you currently have, or have had in the past, any of the following neurological conditions?**

a. Stroke Yes No

b. Concussion Yes No

c. Seizures Yes No

d. Movement disorders Yes No

e. Other: _______________________________________________________________________________

**4. Please indicate the nature of your current occupation (circle one).**

Student Art Science/Engineering Social Science Healthcare

Construction Public Service Law Other:_________________________

**5. Based on personal preference, please rank your enjoyment of Dario Robleto’s exhibit titled ‘The Boundary of Life is Quietly Crossed’ (circle one).**

1 2 3 4 5

I did not enjoy this exhibit I thoroughly enjoyed this exhibit

**6. Please indicate on the following map which piece (if any) you found the most aesthetically pleasing.**


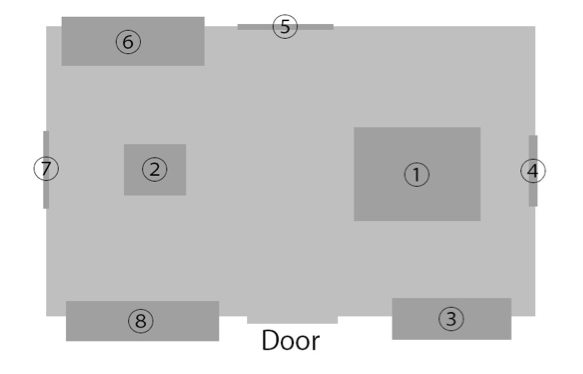


**7. Please indicate on the following map which piece (if any) you found emotionally stimulating and the nature of that stimulation (positive or negative).**


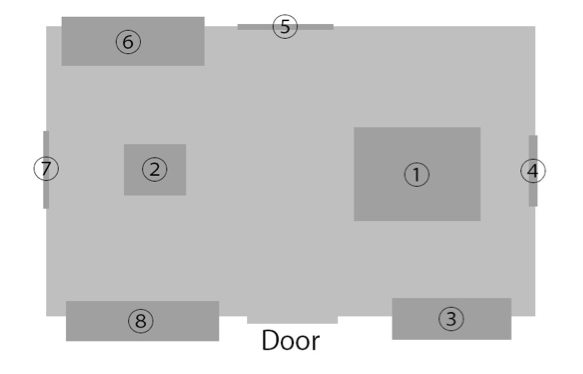


1 2 3 4 5

Negatively stimulated Neutral Positively Stimulated
